# The radiation footprint on the pediatric trauma patient

**DOI:** 10.1186/s12245-018-0175-x

**Published:** 2018-03-14

**Authors:** Raquel M. Schears, Zainab Farzal, Zehra Farzal, Anne C. Fischer

**Affiliations:** 1 The Heller School for Social Policy and Management, Brandeis University, Waltham, MA USA; 2 Department of Otolaryngology, University North Carolina, Children’s Hospital, 101 Manning Drive, Chapel Hill, NC USA; 3 Department of Neurology, MedStar Georgetown University Hospital, Reservoir Rd NW, Washington, DC, 3800 USA; 4 Florida Atlantic University/St. Mary’s Medical Center, 927 45th Street, Suite 301, West Palm Beach, FL 33407 USA

## Abstract

**Background:**

The actual baseline of radiation exposure used in evaluating pediatric trauma is not known and has relied on estimates in the literature that may not reflect clinical reality. Our objectives were to determine the baseline amount of radiation delivered in a pediatric trauma evaluation and correlate radiation exposure with trauma activation status to identify the cohort most at risk.

**Methods:**

We retrospectively evaluated trauma patients (*N* = 1050) at an independent Level I children’s hospital for each level of trauma activation (consults, alerts, stats) from June 2010 to January 2011. Those patients with full dosimetry (*N* = 215) were analyzed for demographics, mechanism of injury, Injury Severity Score, imaging modalities, and total effective radiation dosages during the full trauma assessment from the time of injury to discharge.

**Results:**

Demographics included gender (143 males, 72 females) and average age (5.5 years [range < 1–16]). The most radiation was conferred from CTs and greatest in trauma stats, followed by alerts, then consults (*p* < 0.001 for stat and alert doses compared to consults). Repeated imaging was common: 35% of stats had 2–3 CTs and 40% had 4–10 CTs (range 0–10 CTs). The average non-accidental trauma consult utilized four times as many CTs as the average consult (*p* = 0.002). Most outside hospital CTs (66%) delivered more radiation: 50.0% were at least double the standard pediatric dosage.

**Conclusions:**

This study is the first to identify the actual baseline of radiation exposure for one trauma evaluation and correlate radiation exposure with trauma activation status. Factors associated with highest radiation include stat activations, suspected non-accidental traumas (NAT), and outside hospital system imaging.

## Background

### Introduction

The overuse of ionizing radiation in radiographic imaging is a timely and important topic of discussion in the lay press and the medical community. The era of diagnostic imaging changed significantly with the availability of computed tomography (CT) scans. Beginning in the late 1970s, CT scans became highly preferable given their accessibility and ease of scanning. As a result, there was a surge in the use of CT imaging over the past several decades. The number of CT scans obtained annually in the USA increased approximately 40-fold, from 2 million scans in 1980 to 81.2 million scans by 2014, grossly in excess of the growth of the population [1, 2]. This trend has resulted in increased radiation exposure among patients which is concerning since it is thought that up to 30% of CT scans performed may be unnecessary [3].

### Importance

The link between ionizing radiation and oncogenesis is well known. A hypothetical model based on the cumulative exposure to what was once perceived to be a low and “unconcerning” threshold at the time has now shown that *radiation at all levels* is cumulative across the population [4]. Children are particularly prone to developing malignancies secondary to ionizing radiation for two reasons. First, they are more radiosensitive due to the presence of rapidly dividing cells in their bodies [5]. Second, children have a longer lifespan during which the malignancy may manifest [5, 6]. It is postulated most radiation-induced malignancies are dormant for at least 40 years following exposure [7, 8]. There is growing concern that CT imaging is overused, and it is estimated that up to 2% of all US cancers may now be attributable to the radiation from CT examinations [1, 4]. This was highlighted for the first time in a retrospective cohort review which showed the incidence of 1 excess case of a brain tumor and 1 excess case of leukemia for every 10,000 head CT scans performed among patients under the age of 10 in the 10 years following a single scan [9]. While this study analyzed tumor rates from an epidemiological perspective and not an individual risk per one CT scan, it highlighted the importance of acknowledging the detectable incidence of cancer and the impact of cumulative radiation exposure per child even if only measurable on a population basis. As a result of increased awareness of the potential hazards involved, there is a rising initiative to diminish radiation exposure particularly in children who are most affected by it.

Injury is currently the leading cause of morbidity and mortality among children [10]. In 2010, the leading cause of death in children aged 1–19 years was injury-related according to the CDC [11]. Every year, more than 10 million children are evaluated and treated in emergency departments for traumatic injuries around the USA [12]. As in other patient populations, the use of CT scans has increased in pediatric trauma patients [13]. The high index of suspicion in trauma has created a paradigm of reflexively scanning patients, even those who are asymptomatic, to ensure identifying all potential injuries presumed by the mechanism of injury.

### Goals of this investigation

Our goal was to identify the actual radiation doses used in the evaluation of a pediatric trauma patient from the time of injury since the numbers available in the literature are hypothetical extrapolated doses. Our hypothesis was that trauma activation status is a consistent determination of the acuity of trauma across institutions that best correlates with the amount and type of imaging used to identify potential injuries often presumed by mechanism. We correlated the amount of imaging with trauma activation status in order to identify the amount of radiation per a given activation status to identify the subset of patients most at risk for higher exposures and the subset most amenable to the consideration of reduced imaging strategies. We chose a time period (1) that pre-dated the induction of many reiterative and variable changes to reduce radiation such as newer CT modalities, as flash CT scans, and implementation of pediatric-specific lower dose scanning protocols and (2) when documentation of the dosimetry was first available for collection to obtain the most realistic level of exposure, given the serious epidemiologic implications for the future.

Most studies [14–16] have focused on a mean effective dose for the overall population, averaging all levels of trauma acuity. Institutions vary from each other in respect to their trauma acuity, and therefore, this overall averaging on estimations in the literature underestimates the radiation dose for stat patients and overestimates exposure in non-urgent trauma consult patients.

## Methods

### Study design and setting

Our study was an Institutional Board Review (IRB)-approved retrospective review of the trauma registry at Children’s Medical Center in Dallas, an independent Level I children’s hospital. We evaluated the full encounter from time of injury, including initial outside hospital evaluation for transferred patients, in all trauma activations (*N* = 1050) which included stats, alerts, and consults from June 1, 2010, to January 31, 2011. This time interval represented the earliest time period during which complete dosimetry was initially captured at our hospital prior to the multitude of iterative and variable changes introduced through quality improvement in imaging to reduce radiation exposure.

### Selection of patients and outcomes

The trauma population at Dallas Children’s Medical Center was stratified into the standard three activation statuses based on specific criteria (Table 1). Briefly, trauma consults reflect the lowest activation level, followed by trauma alerts which are triaged at a greater level of concern, while trauma stats represent the highest activation level with potentially life-threatening injuries. This classification is based on the American College of Surgeons Committee on Trauma recommendations [12]. Of the 1050 patients in the 7-month time frame, 739 were admitted to the inpatient service. A total of 60 trauma stat patients with available dosimetry were seen in this time period. Due to a disproportionately higher volume of trauma consult and alert patients, only those presenting in the first 2 months were included for comparison (alerts *N* = 85, consults *N* = 70) to approximate the numbers in the trauma stat subset. Two hundred fifteen patients met inclusion criteria (after the exclusion of 32 patients with missing outside hospital (OSH) imaging) with complete dosimetry available from the time of injury to trauma evaluation on transfer and hospitalization. These patients’ records were analyzed for demographics, mechanism of injury, Injury Severity Score (ISS), imaging modalities, total radiation exposure including the number of CT scans and plain films, and CT radiation dosages in milliSieverts (mSvs). All on-site imaging including the number of CT scans, plain films, and CT doses were taken directly from the Children’s Medical Center radiology records.

**Table 1:** Children’s Medical Center Dallas trauma activation criteria

Trauma Stat
Traumatic cardiopulmonary arrest from penetrating trauma
Traumatic injury with signs of shock
Penetrating injuries to the head, neck, chest, abdomen or pelvis (excludes lacerations in the stable patient)
Respiratory distress secondary to trauma, respiratory compromise/obstruction and/or intubation on scene
Neurological injury with a GCS equal to or less than 8 without sedation
Suspected spinal cord injury: associated with flaccidity, are flexia or unexplained hypotension
Crush or Amputation proximal to the wrist or ankle with signs of shock
Any trauma transfer with respiratory and/or hemodynamic instability and/or GCS equal to or less than 8 without sedation or paralytics and/or patients receiving blood to maintain vital signs
Any intubated trauma transfer
Emergency physician’s discretion
Trauma Alert
Traumatic cardiopulmonary arrest from blunt trauma
Motor Vehicle Crashes (includes ATV’s) with reported history of: ejection of the patient from the vehicle, prolonged extrication (> 20 minutes), a rollover collision, death of an occupant in same vehicle
Neurological injuries with a GCS of 9 to 14
Hanging or strangulation mechanisms
Auto-Pedestrian or Auto-Bike Crashes involving speeds equal to or greater than 20 mph
Falls greater than 2nd story or 20 feet
Bilateral femur fractures or 3 or more long bone fractures
Crush injuries to chest or abdomen
Crush or Amputation injuries proximal to the wrist or ankle in the stable patient with fracture or significant tissue loss
Significant lacerations to head or neck in the stable patient - Lacerations that are deep or with significant tissue loss
Any transfer with a grade IV solid organ injury or two or more solid organ injuries
Trauma Consult
Child abuse cases to be admitted
Any trauma related injury where two or more systems are involved
Any patient that has a single system injury that requires admission and the mechanism is an MVC, MPC, ATV

Being a Level I trauma service, our trauma admissions included patients transferred to our institution’s emergency department after a prior evaluation at an outside hospital. Patients transferred from an outside hospital who were included in the study had imaging and radiation data previously uploaded into our hospital’s records for a total of 61 outside CT scans. Transferred patients with no recorded radiation dose in our electronic medical records were excluded from the study.

### Radiation dose measurements

In order to calculate effective radiation doses in milliSieverts, precise DLP (dose-length product) measurements were taken for each CT scan performed from Children’s Medical Center radiology records. A dose-length product is a measure of radiation exposure due to CTs which takes both the amount of radiation used as well as the length of the CT scan into account [17]. The DLP value was multiplied by conversion factors provided in the literature by Shrimpton et al [18] to adjust for patient age and provided the final effective radiation doses in milliSieverts. While several sets of conversion factors exist, this is the most widely used method and was thus utilized in our study [19].

### Statistical analysis

Student’s *t* tests were used to determine the statistical significance of (a) radiation dose from CT scans and (b) number of CT scans performed in the subgroups compared to the trauma consults. The level of significance was set at *p* < 0.05. To avoid introducing a confounding variable in the trauma cohorts, non-accidental traumas (NATs) were examined as a distinct category. Of note, no statistical analysis was done for the subcategory of NAT alerts due to an *N* of 2.

## Results

### Characteristics of study subjects

The demographics, Injury Severity Scores (ISSs), and mechanisms of injury comparing consults, alerts, and stats are presented in Table 2. Most trauma consults were due to fractures from falls. Among alerts, the majority of patients presented due to car accidents or falls while the stats were mostly either car accident injuries or NATs.

**Table 2:** Study population characteristics

Demographics	Consults	Alerts	Stats
Age	Median 5.5 years [< 1, 16]		
Gender	143 males, 72 females		
ISS	7.7 [1, 16]	8.8 [1, 17]	17 [3, 31]
Mechanisms of injury			
Fall	37	11	9
MVC	9	43	19
MVC-pedestrian	1	27	7
NAT	9	2	12
Sports injury	5	0	3
Struck with object	8	6	6
Animal bite	4	3	1
GSW	2	0	3
Bike accident	0	9	2
Other	5	9	1

*ISS* Injury Severity Score, *MVC* motor vehicle collision, *NAT* non-accidental trauma, *GSW* gunshot wound

### Main results

On initial analysis, it was evident the NAT cohort as a group was exposed to more radiation than other trauma patients. To avoid inflating the average radiation doses for each activation status based on the number of NATs in each subcategory, we analyzed radiation exposure per patient in two subsets: (1) trauma patients excluding NATs and (2) NATs for all three activation statuses.

### Trauma patients excluding NATs

Trauma stat patients received the most radiation followed by alerts when compared to trauma consults for CT dose and total number of CT scans (*p* < 0.001) (Table 3). The increase in radiation dose positively correlated with higher activation status and is best exemplified by the CT dose factor (Table 3). The CT dose factor represents the relative radiation due to CT scans as a factor of the CT dose for consults (0.79 mSv). A 10-fold increase between consults and stats was demonstrated by the CT dose factor.

**Table 3:** Radiation exposure per trauma patient

Activation status	Number	CT/patient*	CT/patient *p* value	CT dose (mSv) *†	CT dose factor	CT dose *p* value	X-rays/patient
Trauma patients excluding non-accidental traumas (NATs)							
Consults	61	0.3	N/A	0.79 ± 2.2	1	N/A	5.5
Alerts	83	1.6	< 0.001	5.34 ± 6.6	6.76	< 0.001	8.4
Stats	48	2.4	< 0.001	8.00 ± 8.4	10.13	< 0.001	13.7
NATs							
Consults	9	1.2	0.002	2.83 ± 1.8	3.58	0.009	28
Alerts	2	1	N/A	3.28 ± 1.5	4.15	N/A	27.5
Stats	12	3	< 0.001	9.19 ± 5.0	11.63	< 0.001	38.7

*p* values were computed for each group relative to trauma consult patients. *T* tests were not performed comparing NAT alerts with trauma consults due to small sample size

*All values are averages

†All radiation doses include ± SD and are calculated in milliSieverts (mSv)

### Non-accidental traumas

**Fig. 1:**
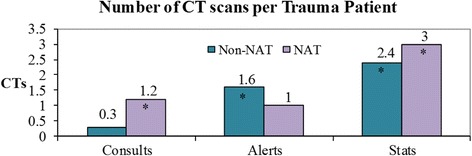
Number of CT scans per trauma. *Groups that were found to have statistically significant increases in the number of CT scans relative to the number of CTs performed in trauma consults. Note: A *T* test was not performed comparing NAT alerts with trauma consults due to small sample size

Overall, NAT evaluations had more imaging. NAT consults and stats had more CT scans than their non-NAT counterparts. In fact, the average NAT consult was subject to four times as many CTs as the average trauma consult (Table 3, *p* < 0.05). The number of CTs generally increased with activation status (Fig. 1 and Table 3), although the number of CTs for NAT alerts (1.0) was similar to CTs per NAT consult (1.2). Total CT dose per patient (Table 3) increased with higher activation status. Specifically, NAT stats had a CT dose factor close to 12.

### CT use across entire cohort

#### Repeated imaging

Analyzing imaging patterns in the entire study population, the greatest variation in the number of CTs was noted among the stats; number of CTs ranged from 0 to 10 with 2–3 CTs performed in 35% of the stat patients and 4–10 CTs in 40% of the stat patients per evaluation. The studies most often repeated across all activation statuses were head CTs, face/sinus CTs, and neck CTs with repetition rates of 45, 13.8, and 10% respectively.

#### Dosing per scan

Sixty-six percent (40/61) of outside CT scans delivered more radiation than the pediatric protocol CT scans, of which 50.0% of the doses were at least double the standard pediatric dosage delivered at the children’s hospital. A subset analysis of all patients who received OSH imaging (*N* = 30) was performed (Table 4). The highest average OSH CT dose was noted in alerts (9.77 mSV), followed by consults (7.97 mSv), and with stats having the lowest dose (4.77 mSv). Analysis of different CT studies was not performed due to insufficient numbers for meaningful results in each separate category of CT scans.

**Table 4:** Outside hospital imaging

	Consults	Alerts	Stats
No. of patients with OSH imaging	10	14	16
Average total OSH CT dose (mSv)	7.97	9.77	4.77

### Plain films

An increase in the number of plain films per patient with higher activation status was noted in both general trauma patients and NATs. NAT alerts (27.5 plain films) were typically similar to NAT consults (28 plain films) (Table 3). The majority of these plain films in NAT patients are accounted for by the routine ordering of a skeletal survey which typically consists of 23–24 plain films. Most plain films in addition to the first 23–24 films comprised repeat full body scans or were follow-up plain films of body regions where injuries were highly suspected or identified.

### Limitations

There were several limitations in this study. Firstly, the mean effective radiation doses calculated were adjusted with conversion factors dependent on patient age and an average body weight for that age. As a result, the doses provided may overestimate the doses in underweight patients and underestimate doses in overweight or obese patients. However, all current studies are held to this limitation. Additionally, different sets of conversion factors exist in the literature for determining mean effective dose with all resulting in slightly different dosage values [16] but are grossly similar. Alternative calculation methods may report values to be as much as 10.2 ± 10.1% lower to 28 ± 37.3% higher than the method used in this study [19]. Hence, the specific method used must be reported by each study in order to correctly compare the results across studies. Since our study was retrospective, another key limitation was the inability to definitely determine why some of the patients were re-scanned. Although it may be evident for some that clinical deterioration was the main cause based on medical record notes, it is difficult to differentiate between some causes such as poor scan quality of an OSH scan, redundant scanning given lack of accessibility of outside imaging, as opposed to lost/un-transferred disks in a retrospective analysis.

## Discussion

Pediatric trauma patients encompass a population particularly prone to radiation exposure, given their increased susceptibility to ionizing radiation, and often receive care at non-pediatric hospitals which may not have attention to pediatric protocol dosing. Given the broad acceptance of non-operative management in trauma, there is a reasonable concern whether such widespread use of advanced imaging is warranted in all pediatric patients, particularly since surgical decision-making and interventions are now primarily driven by the physiologic status of the patients. Specifically, studies have shown that CT scans rarely influence the decision for operative intervention in trauma patients such as those who have sustained blunt abdominal trauma [20]. In patients who have been managed non-operatively, CT scans have shown limited value in directing further management and follow-up [21].

While an increase in imaging with a higher activation status appears intuitive, the CT dose factor reflecting the 10-fold CT dose increase between general trauma consults and general stats and a similar near 12-fold increase between general consults and NAT stats are both completely unexpected and grossly exceeded our expectations. A National Academy of Science report has estimated that children aged 15 and below have a 40% increase in cancer rate with exposures in the range of 10 to 20 mSv [22]. Twenty-one percent of our trauma alerts and stats received doses within this range for a *single* trauma evaluation which is a very important baseline determination for future epidemiologic analysis. Furthermore, children with multiple trauma evaluations would be at even higher risk over time. The sharp increase in radiation exposure with activation status may be a result of (i) a higher index of suspicion in higher activation statuses, (ii) imaging decisions based on the presumption of injuries by mechanisms, and (iii) the pressure to identify all injuries expeditiously in the initial trauma evaluation.

Identifying the actual radiation doses are important to determine the actual exposures our population of children is receiving. Prior trauma studies have analyzed average radiation exposure *based on estimations* averaged across all levels of traumas extrapolated from the literature. The largest study queried the NTDB in 2010 included 84,863 patients and showed the mean effective CT radiation dose to be 12.0 mSv^15^; however, the radiation doses were not based on actual radiologic records and were actually extrapolated from the literature. Also with such huge numbers of traumas that are not differentiated out by level of severity, it is easy to recognize that the simple trauma consults or low grade traumas would outnumber the more severe ones and thus power this finding to underestimate the amount of imaging a moderate or severe trauma work-up needs. Clearly with one trauma being 12 mSV, then, a child with just one trauma evaluation would potentially be in the concerning zone of cumulative radiation exposure. Our study shows that the real exposure is much higher for a stat activation so averaging all trauma activations may not give a clear accounting of which traumas actually receive a concerning amount of radiation. The National Survey of Children’s Health (NSCH) has reported that 22.6% of children have over two serious trauma evaluations so the impact of higher radiation dosing on top of multiple trauma evaluations has serious epidemiologic consequences [23]. Additionally, the study [15] excluded patients who were not imaged within the first 24 h of admission and did not account for (i) those who were imaged subsequently and (ii) the less worrisome traumas who were not imaged at all, as opposed to our study which included the entirety of imaging for all patients during the full encounter including OSH imaging.

The subset analysis of NAT patients in our study provided a focus on a less studied patient group. A recent study has shown that 6.2 out of 100,000 children under the age of 18 have endured severe physical abuse [24]. Furthermore, this rate is nine times higher in patients under the age of one [24]. Since these patients suffer polytrauma, they are particularly prone to more frequent trauma evaluations and more extensive imaging compared to the corresponding cohort in the same activation status. The reason for extensive imaging appears bipartite. First, given the higher likelihood of multiple injuries, the pre-existing high level of suspicion in trauma patients is further justified. Second, positive imaging findings serve as evidentiary proof in investigations once legal action is pursued. While a recent study has stressed the importance of surgical evaluations in non-accidental traumas [25], a detailed analysis of radiation exposure in this subset has yet to be conducted.

Additionally, it is important to understand the significance of the skeletal surveys performed in NAT patients which utilizes 23–24 plain films. The amount of exposure is diminishingly small since each plain film of the abdomen/pelvis accounts for a mean effective dose of only 0.015 mSv in a newborn to 0.05 mSv in a 15-year-old [26]. While X-ray scans impart significantly less radiation than CT scans, their high utilization demonstrates the comprehensive nature of imaging to prove a NAT case for legal intervention.

An advantage of our study was the inclusion of outside hospital (OSH) imaging which was uploaded to our imaging database. As a result, we were able to note the incidence of repeated imaging upon transfer and compare actual outside hospital radiation dosing with pediatric dosing at our pediatric center. Although overall and children’s hospital CT doses correlated with activation status, we found that OSH CT doses *did not* have a similar correlation. Instead, trauma alerts received the most radiation followed by consults and then trauma stats. Alerts may have received the most radiation as OSH physicians may have viewed them as a group of patients just stable enough to be managed without specialist consultation at a children’s hospital. As a result, they likely performed a complete work-up and later realized they needed to transfer the patients to a children’s hospital. Presumably, the lowest CT dose in the trauma stats group is likely related to the realization that the patient should not be managed at the OSH and required transfer soon after the initial images were obtained.

The redundancy in imaging upon transfer was also shown in a prior study in which a high incidence of repeated CT scans in transferred trauma patients along with higher hospitalization costs were reported [27]. This is particularly concerning since the reason for re-imaging in these patients reflects the lack of coordination between hospitals and timely access to transferred imaging studies. ED/trauma staff must advocate for the establishment of timely communication between major trauma centers and outside hospitals to reduce the delivery of excess radiation.

Our study had the additional advantage of evaluating the full hospital stay as opposed to initial evaluations limited to the first 24 h. There is currently no data in the literature explicitly reflecting the propensity of repeated imaging of admitted trauma patients over the entire hospital stay. Our study showed that patients who received multiple CT scans as well as repeat full body plain film scans were often those who were hospitalized.

A prior study by Groner et al., [28] has suggested that traumas worked up by an emergency medicine attending physician are less likely to receive excessive radiation compared to an on-call trauma surgery resident who has less experience and is not constantly present with the patient. While this study highlighted only moderate level traumas at the authors’ institution, such a model may benefit lower and higher activation statuses as well. As such, we advocate further education of surgical trainees and the new trauma model [28] that proposes that trauma surgeons should play the role of consultants rather than primary responders. Our study highlights the importance of adherence to the principle of maintaining radiation exposure “as low as reasonably achievable” (ALARA) [29]. The ALARA principles involve four cornerstones to decrease radiation dosimetry: (1) use weight-based protocols, (2) consider alternative non-radiating modalities, (3) use focused or limited-view studies when clinically appropriate, and (4) dissuade repeat CT studies.

These strategies advocate low-dose pediatric-specific protocols and other techniques such as flash CT, minimizing thin-cut CT imaging, utilizing “justification and optimization” determinations in granting CT requests (to avoid additional or unnecessary radiation exposure), and dedicated pediatric CT imaging services with pediatric-specific CT technologists to improve compliance with adjusted lower CT exposure parameters and lower estimated effective doses of radiation delivered to pediatric patients. These ALARA strategies have resulted in much greater compliance with pediatric dose-adjusted CT protocols and well-recognized reduced radiation exposure to patients.

The majority of pediatric trauma patients are managed at non-pediatric (adult) trauma hospitals, since children are quickly transported to the nearest hospital initially for evaluation and thus are exposed to twice the radiation dose that they would have been exposed to in a pediatric hospital with a pediatric CT imaging dose reduction protocol in effect [30]. Moreover, in an age during which the paradigm of “pan-scanning” is common practice, we must enforce protocols to minimize radiation exposure in our patients. For this to occur, the entire multidisciplinary team including ED staff, pediatric/trauma surgeons, and radiologists must be on board. 

## Conclusions

In summary, this study is the first to correlate the degree of radiation exposure with trauma activation status defining actual average radiation exposure values for each subset. The identified factors associated with the most radiation include stat activations, suspected NATs, and trauma patients transferred after OSH imaging due to tendency to use adult dosing parameters on pediatric trauma patrons. To minimize the radiation footprint, we may need to change the current practice of widespread utilization of advanced imaging to identify all possible injuries regardless of symptoms.
